# Collaboration Between Nurses and Patients’ Families in Managing Chronic Heart Failure in Older Adults: A Qualitative Study

**DOI:** 10.3390/healthcare14070853

**Published:** 2026-03-27

**Authors:** Abdulaziz M. Alodhailah, Albandari Almutairi, Thurayya Eid, Rayhanah R. Almutairi, Asrar S. Almutairi, Ashwaq A. Almutairi, Waleed M. Alshehri, Bader M. Almutairy, Faihan F. Alshaibany

**Affiliations:** 1 Department of Medical-Surgical Nursing, College of Nursing, King Saud University, Riyadh 11451, Saudi Arabia; 2 Department of Maternal and Child Health Nursing, College of Nursing, King Saud University, Riyadh 11451, Saudi Arabia; alafi@ksu.edu.sa; 3 Community and Psychiatric Mental Health Nursing Department, College of Nursing, Princess Nourah bint Abdulrahman University, Riyadh 11671, Saudi Arabia; 4 Monash Nursing and Midwifery, Monash University, Melbourne, VIC 3800, Australia; 5 Department of Community and Mental Health Nursing, College of Nursing, King Saud University, Riyadh 11451, Saudi Arabia; 6 Department of Nursing Administration and Education, College of Nursing, King Saud University, Riyadh 11451, Saudi Arabia

**Keywords:** chronic heart failure, older adults, primary healthcare, family caregivers, nurse–family collaboration, qualitative research, Saudi Arabia, phenomenology

## Abstract

**Background**: Chronic heart failure (CHF) in older adults requires sustained self-management and close follow-up, yet day-to-day care is often carried out by families with support from primary healthcare nurses. In Saudi Arabia, where family caregiving is culturally normative, collaboration between nurses and patients’ families may be pivotal to effective CHF management, but remains insufficiently understood in primary healthcare contexts. **Methods**: A qualitative study informed by an interpretive phenomenological approach was conducted. Participants (*n* = 24; 12 nurses and 12 family caregivers) were recruited using purposive sampling from primary healthcare centers in Riyadh, Saudi Arabia. In-depth, semi-structured interviews were conducted in Arabic or English, audio-recorded, transcribed verbatim, and analyzed using reflexive thematic analysis following Braun and Clarke’s six-phase framework. Strategies to enhance trustworthiness included member checking, peer debriefing, maintenance of an audit trail, and reflexive journaling. **Results**: Twenty-four participants (12 nurses and 12 family caregivers) were interviewed. Four interrelated themes were generated from both nurses’ and family caregivers’ accounts. (1) “We Are Caring Together”: Collaboration was experienced as shared responsibility for daily CHF management, grounded in trust; (2) Navigating Roles and Boundaries: Participants described unclear expectations, role overlap, and tension between professional authority and family knowledge; (3) Communication as the Engine of Collaboration: Effective partnerships depended on clear information exchange, caregiver-tailored education, and continuity of contact, while communication gaps created uncertainty and delayed support-seeking; and (4) Cultural and System Constraints Shaping Collaboration: Strong family obligation motivated caregiving but also intensified moral pressure and limited help-seeking, while time pressure and fragmented services constrained meaningful engagement and continuity across settings. **Conclusions**: Nurse–family collaboration in CHF management is relational, shaped by trust, role negotiation, and communication, and constrained by cultural norms and system pressures. This study contributes to the literature by demonstrating how moral obligation, hierarchical professional norms, and system fragmentation distinctively shape collaboration in the Saudi primary care context, extending existing conceptualizations derived primarily from Western individualist settings. Strengthening collaboration requires explicit role clarification, health literacy–informed caregiver education, continuity of contact, and organizational supports. Findings are limited by purposive sampling, single-city context, and exclusion of patient perspectives.

## 1. Introduction

Chronic heart failure (CHF) is a leading cause of morbidity, hospitalization, and mortality among older adults worldwide. As populations age and survival following cardiovascular events improves, the prevalence of CHF continues to rise, placing substantial demands on healthcare systems and long-term care infrastructures [1]. Older adults living with CHF often experience complex symptom burdens, including dyspnea, fatigue, fluid retention, cognitive changes, and reduced functional capacity, which require continuous monitoring and multifaceted management strategies [2]. These challenges are further compounded by multimorbidity, polypharmacy, and age-related physiological decline, making effective long-term management particularly demanding in this population [3,4].

Contemporary models of CHF care emphasize self-management as a cornerstone of disease control, aiming to reduce symptom exacerbation, prevent avoidable hospital admissions, and improve quality of life [5]. Self-management typically includes medication adherence, dietary regulation, fluid restriction, symptom recognition, and timely healthcare seeking [6]. However, for many older adults, the ability to independently engage in these behaviors is limited by physical frailty, cognitive impairment, sensory deficits, and social vulnerability [7]. As a result, family members frequently assume critical roles in supporting daily care activities, decision-making, and care coordination [8].

Families of older adults with CHF often act as informal caregivers, assisting with medication management, symptom monitoring, appointment scheduling, and lifestyle modifications [9]. While this involvement can enhance continuity of care and promote adherence to treatment regimens, it may also generate caregiver burden, emotional strain, and role conflict, particularly when expectations are unclear or support is insufficient [10]. Effective collaboration between healthcare professionals, especially nurses, and patients’ families is therefore essential to ensure that caregiving responsibilities are shared, supported, and sustainable.

Nurses occupy a central position in CHF management across acute, outpatient, and community settings. Their roles extend beyond clinical monitoring to include patient and family education, psychosocial support, care coordination, and transitional care planning [11]. In geriatric CHF care, nurses are often the primary point of contact for both patients and families, positioning them uniquely to facilitate collaborative partnerships [12]. Evidence suggests that nurse-led interventions that actively involve family caregivers can improve symptom control, enhance self-care behaviors, and reduce rehospitalization rates [13]. Nevertheless, the quality and nature of nurse–family collaboration vary widely depending on institutional structures, communication practices, cultural norms, and professional boundaries [14].

Despite recognition of the importance of family involvement, collaboration between nurses and families is not always straightforward. Families may feel excluded from decision-making, inadequately informed, or insufficiently prepared to manage complex care tasks at home [15]. Conversely, nurses may experience tensions related to time constraints, unclear caregiver roles, differing expectations, or concerns about family members’ competence and emotional readiness [16]. In CHF care, where management frequently transitions between hospital and home, these challenges can undermine continuity, safety, and patient outcomes [17]. Transitional periods are particularly vulnerable to communication breakdowns and fragmented responsibility [18], making effective nurse–family collaboration critical to ensuring accurate information transfer, consistent care practices, and early identification of symptom deterioration [19]. However, much of the existing literature has focused on clinical outcomes or caregiver burden, with less attention given to the relational and experiential dimensions of collaboration as perceived by nurses and families themselves [20].

Qualitative research offers valuable insights into the lived experiences, meanings, and relational dynamics that shape healthcare practices [21], and previous qualitative studies in chronic illness contexts have highlighted the importance of trust, mutual respect, shared understanding, and flexible role negotiation in sustaining effective nurse–family partnerships [22]. Yet qualitative evidence examining nurse–family collaboration specifically in CHF primary care within collectivist cultural contexts, such as Saudi Arabia, remains scarce.

Therefore, the aim of this qualitative study is to explore collaboration between nurses and patients’ families in the management of chronic heart failure among older adults. By examining the perspectives of both nurses and family caregivers, this study seeks to illuminate the challenges and facilitators of collaborative care in CHF management. Specifically, this study draws on relational coordination theory [23,24], professional boundary theory [25], and health literacy frameworks [26,27] to interpret how collaboration is shaped by trust, role negotiation, communication practices, and cultural context within Saudi primary healthcare. By situating findings within these conceptual perspectives from the outset, the study aims to move beyond descriptive accounts toward a theoretically informed understanding of nurse–family collaboration. In-depth insights into these experiences can contribute to the development of more inclusive, responsive, and family-centered nursing practices for older adults living with chronic heart failure.

## 2. Methods

### 2.1. Design

This study adopted a qualitative design informed by interpretive (hermeneutic) phenomenology to explore how nurses and family caregivers experience and negotiate collaboration in managing chronic heart failure (CHF) among older adults in primary healthcare settings. Interpretive phenomenology, grounded in the tradition of Heidegger and Gadamer, emphasizes that understanding lived experience is inherently interpretive, and meaning is actively constructed through dialogue between researcher and participant within specific historical, cultural, and relational contexts [1]. This approach is well suited to exploring how nurses and families make sense of collaboration within Saudi family structures where caregiving norms are strongly influenced by intergenerational obligations and sociocultural values.

Braun and Clarke’s reflexive thematic analysis [23] was employed as the analytic method. This integration was deliberate: thematic analysis offers a systematic procedure for identifying patterns of meaning across narratives, while remaining compatible with phenomenological attention to lived experience [21,22,23]. The design privileged rich, first-person narratives to access accounts of how collaboration is enabled, constrained, or reshaped across care encounters and transitions. The research process adhered to the Standards for Reporting Qualitative Research (SRQR) to enhance methodological transparency and interpretive rigor [16].

### 2.2. Study Setting and Recruitment

This study was conducted in five governmental primary healthcare centers (PHCCs) in Riyadh, Saudi Arabia. The centers were purposively selected to ensure variation in service delivery, patient populations, staffing patterns, and local contexts. Riyadh is a large metropolitan city with diverse socioeconomic communities, and its primary healthcare system provides comprehensive services for patients with chronic conditions, including chronic heart failure (CHF), such as follow-up care, medication management, health education, and referral services. PHCCs play a central role in ensuring continuity of care for older adults with CHF.

A purposive sampling strategy was used to recruit participants with direct experience in CHF management. In qualitative research, purposive sampling is appropriate for obtaining rich, in-depth data from individuals with relevant experience. However, this approach may introduce selection bias, as participants who agree to take part may be more engaged or have more positive experiences. In addition, recruitment through PHCCs may limit the inclusion of less-engaged caregivers. The urban setting may also limit the transferability of findings to other contexts. These issues are further addressed in Section 4.2.

Data collection and analysis were conducted concurrently, and recruitment continued until thematic saturation was achieved. A total of 24 participants were included in the study, comprising 12 nurses and 12 family caregivers. This sample size is consistent with qualitative research recommendations for phenomenological and thematic studies. Equal representation of nurses and caregivers was intended to capture both professional and family perspectives.

Saturation was assessed iteratively during data collection. After each interview, transcripts were reviewed and coded to identify emerging themes. A saturation log was maintained to track the development of themes across interviews. Saturation was considered achieved when no new themes or meaningful insights emerged in consecutive interviews. This occurred after interview 22, and two additional interviews were conducted to confirm saturation.

Participants were recruited based on predefined inclusion and exclusion criteria. Eligible nurses were registered nurses working in PHCCs in Riyadh with at least one year of experience in primary care or chronic disease clinics and who were involved in the care of older adults (≥60 years) with CHF. Eligible family caregivers were adults (≥18 years) providing unpaid care for at least six months to an older adult with CHF and who had attended at least one PHCC visit or communicated with PHCC nurses. All participants were able to complete an interview (45–90 min) in Arabic or English and provided written informed consent. Individuals with cognitive impairment or severe psychiatric illness, those unable to provide informed consent, caregivers providing only temporary support, and nurses without CHF-related responsibilities were excluded.

### 2.3. Data Collection

Data were collected through in-depth, semi-structured interviews covering communication patterns, perceived roles, expectations, challenges, and strategies for shared management. Interviews were conducted in private rooms within participating PHCCs for nurses and in a private setting selected by family caregivers (either within the PHCC after an appointment, or via secure remote interview when attendance was not feasible).

To minimize social desirability bias and perceived power differentials, interviews were conducted by a researcher with doctoral-level qualitative training and over six years of experience in health services research. The interviewer was not involved in participants’ clinical care or PHCC management. Field notes were documented immediately after each interview to capture contextual information, non-verbal cues, and reflections relevant to interpretation.

### 2.4. Interview Structure and Conduct

The interview guide was developed iteratively based on the study aim, primary care CHF management pathways, and literature on family involvement and collaborative care. It was reviewed by qualitative experts and piloted with two participants (one nurse and one caregiver) to ensure clarity and cultural appropriateness; pilot interviews were not included in the final analysis. Open-ended prompts included those given below.

#### 2.4.1. For Nurses

- “Can you describe how you work with families when managing older adults with chronic heart failure?”
- “What does ‘good collaboration’ look like in your daily practice?”
- “What challenges arise when families and nurses have different expectations about care?”
- “Can you share an example of a situation where collaboration improved outcomes, or where it broke down?”

#### 2.4.2. For Family Caregivers

- “How do nurses at the PHCC involve you in managing your relative’s heart failure?”
- “What information or support do you need from nurses to care confidently at home?”
- “When do you feel included in decisions, and when do you feel excluded?”
- “What makes communication with nurses easier or more difficult?”

Probing questions (e.g., “Can you tell me more?” “What happened next?” “How did that make you feel?”) were used to deepen accounts and clarify meanings. Interviews lasted 45–92 min (average approximately 60–70 min). Data collection and early analysis proceeded concurrently to refine probes and ensure adequate exploration of emerging concepts.

### 2.5. Recording and Transcription

All interviews were audio-recorded with participant consent and stored on password-protected, encrypted devices accessible only to the research team. Interviews conducted in Arabic were transcribed verbatim; selected excerpts were translated into English using forward translation, independent review, and reconciliation to preserve meaning. Transcripts were checked against recordings for accuracy and de-identified during transcription, and participants were assigned codes (e.g., N01–Nxx; C01–Cxx) to ensure confidentiality.

### 2.6. Data Analysis

Data were analyzed thematically using Braun and Clarke’s six-phase framework [23], supported by NVivo software (Version 14; QSR International Pty Ltd, Melbourne, Australia). Analysis proceeded as follows: (i) familiarization through repeated reading of transcripts and field notes; (ii) generation of initial codes capturing meaningful units related to collaboration, roles, communication, barriers, and facilitators; (iii) clustering codes into candidate themes (e.g., negotiating roles, trust-building, information gaps, cultural expectations, system constraints); (iv) reviewing themes against the dataset to ensure coherence and distinctiveness; (v) defining and naming themes with clear analytic boundaries; and (vi) producing a thematic narrative supported by illustrative excerpts.

Coding was primarily inductive, with initial codes generated directly from participants’ accounts (e.g., “shared responsibility,” “unclear expectations,” “time pressure,” “family duty”) rather than from a pre-existing framework. No formal deductive coding template was applied; however, the researchers’ familiarity with literature on collaborative care and chronic illness management provided sensitizing concepts. Coding was iterative, with ongoing refinement throughout analysis. To enhance rigor, coding was conducted by the lead analyst and reviewed by a second qualitative researcher, with discrepancies resolved through discussion. Emerging themes were discussed within the research team, and participant feedback on preliminary findings was sought to support trustworthiness and interpretive accuracy.

### 2.7. Ethical Considerations

Ethical approval was obtained from the Institutional Review Board (IRB) of King Saud University, and relevant administrative approvals were secured from participating PHCCs (KSU-HE-25-1419). All participants received an information sheet detailing study aims, procedures, voluntary participation, and confidentiality protections. Written informed consent was obtained prior to interviews. Participants were informed of their right to withdraw at any time without penalty and without any effect on employment status (for nurses) or clinical services (for patients/families). Personal identifiers were removed from transcripts, and any potentially identifying contextual details were generalized. Data were stored securely in accordance with institutional data protection policies.

### 2.8. Rigor and Reflexivity

Trustworthiness was established using Lincoln and Guba’s criteria [28]. Strategies included: member checking with select participants regarding thematic accuracy; peer debriefing with external qualitative experts; maintenance of an audit trail documenting decisions and interpretive developments; and reflexive journaling to acknowledge researcher perspectives and potential biases. The lead researcher maintained reflective notes throughout data collection and analysis to monitor how personal assumptions, clinical background, and cultural positioning might influence interpretation.

## 3. Results

### 3.1. Characteristics of Participants

A total of 24 participants (12 nurses and 12 family caregivers) were included in the study (Table 1). Participants were recruited from governmental primary healthcare centers in Riyadh City, Saudi Arabia. Data saturation was assessed through concurrent data collection and analysis and was considered achieved when no new codes, themes, or experiential insights emerged across consecutive interviews; this occurred at the 22nd interview, with two additional interviews conducted to confirm analytic stability.

Nurse participants ranged in age from 26 to 49 years (mean 35.7, SD 6.4), with the majority being female (*n* = 9). Professional experience ranged from 3 to 22 years (mean 10.8, SD 5.9), including 2 to 15 years in primary healthcare settings. Educational backgrounds included diploma (*n* = 4), bachelor’s (*n* = 6), and postgraduate qualifications (n = 2). All nurses were actively involved in chronic disease clinics, providing follow-up care for older adults with CHF, including medication management, symptom monitoring, patient education, and care coordination, and reported frequent interaction with family caregivers.

Family caregivers ranged in age from 29 to 68 years (mean 44.3, SD 9.8), with 7 females. Relationships included adult children (*n* = 8), spouses (*n* = 3), and one sibling (n = 1). Caregiving duration ranged from 1 to 9 years (mean 4.6, SD 2.3). Most caregivers lived with the patient (*n* = 9), while others provided regular support from nearby locations. Care responsibilities included medication management, diet and fluid monitoring, appointment coordination, symptom observation, and communication with healthcare providers, often alongside employment and other family responsibilities.

### 3.2. Overview of Key Findings

Four themes emerged from the analysis (Table 2). All 24 participants described collaboration as involving shared responsibility for daily CHF care; however, its clarity and sustainability varied. All nurses reported relying on family caregivers to implement care recommendations at home, yet only 8 (67%) described explicitly discussing role expectations. Similarly, 7 caregivers (58%) reported learning their roles through trial and error rather than structured guidance.

All participants identified communication as central to collaboration; however, 9 nurses (75%) reported time constraints limiting education, and 8 caregivers (67%) described unresolved questions after clinic visits. All caregivers described their role as a moral obligation, and 9 (75%) reported reluctance to express difficulty. All nurses and 10 caregivers (83%) identified time pressure and system fragmentation as key constraints on collaboration.

The four themes were: (1) “We Are Caring Together”, shared responsibility and trust; (2) Navigating Roles and Boundaries, unclear expectations and overlapping roles; (3) Communication as the Engine of Collaboration, information exchange, education, and accessibility; and (4) Cultural and System Constraints Shaping Collaboration, family obligation, time pressures, and fragmented services. Table 2 provides further detail.

#### 3.2.1. “We Are Caring Together”

This theme captures how collaboration was experienced as a shared, relational practice rather than a set of discrete tasks. Participants described CHF management as requiring ongoing coordination across clinic and home settings. While clinical responsibility formally rested with healthcare professionals, day-to-day management was largely enacted by families, creating an interdependent model of care. Collaboration was shaped by how responsibilities were distributed and by the trust established between nurses and caregivers.

Nurses described families as “extensions of care” beyond the clinic, while caregivers viewed nurses as anchors providing guidance and reassurance. When collaboration functioned well, participants described collective ownership over the patient’s well-being. However, this required continual negotiation and was vulnerable to breakdown when roles or expectations were unclear.

1. Shared Responsibility for Daily Management

Participants consistently emphasized that managing CHF in older adults depended on shared responsibility, particularly for tasks that extended into everyday life. Nurses described relying on family caregivers to translate clinical advice into sustained action, monitoring symptoms, ensuring medication adherence, regulating diet and fluid intake, and recognizing early signs of deterioration.

*“We can explain everything in the clinic, but the real work happens at home. The family is the one watching the swelling, the breathing, the weight.”*

(N03)

Caregivers echoed this perspective, describing themselves as the primary managers of daily care, yet emphasized that their confidence depended heavily on nurses’ guidance. Many caregivers described a sense of accountability not only to the patient but also to the nurse, framing their role as part of a joint effort rather than an isolated burden.

*“I feel like I am responsible, but not alone. When the nurse explains clearly, I feel we are doing this together.”*

(C07)

However, shared responsibility was not always evenly negotiated. Some caregivers reported assuming extensive responsibilities without explicit discussion, particularly when patients were frail or had limited health literacy. In these cases, caregivers described learning through trial and error, which sometimes generated anxiety and fear of making mistakes.

*“Sometimes I’m not sure if I’m doing it right, especially with the medicines. I wish we could talk more about who does what.”*

(C02)

Nurses acknowledged this tension, noting that time constraints often limited their ability to clarify roles fully, even when they recognized the importance of doing so. As a result, shared responsibility was sometimes implicit rather than explicitly agreed upon, leaving room for misunderstanding and stress.

2. Trust as the Foundation of Partnership

Trust emerged as the cornerstone of effective collaboration, shaping how responsibilities were shared and how openly concerns were communicated. Caregivers described trust as developing gradually through consistent interactions, respectful communication, and nurses’ willingness to listen and respond to their observations.

*“When the nurse listens to what I see at home, I feel respected. Then I trust her advice more.”*

(C04)

Nurses similarly emphasized that trusting relationships enabled more meaningful collaboration. When caregivers were perceived as attentive and committed, nurses felt more comfortable relying on their reports and engaging them as active partners in care decisions.

*“You know which family you can depend on. When there is trust, you take their words seriously.”*

(N11)

Conversely, when trust was weak or absent, collaboration became fragile. Some nurses expressed concern about caregivers misunderstanding instructions or selectively following advice, while caregivers reported hesitancy to ask questions when they felt judged or rushed.

*“If the nurse seems busy or annoyed, I just stay quiet, even if I’m worried.”*

(C09)

Trust was therefore not static but continuously shaped by everyday interactions. Participants described it as something that could be strengthened through continuity of care, clear communication, and acknowledgment of each other’s roles, or eroded by time pressure, dismissive attitudes, or inconsistent messages. Ultimately, trust functioned as the relational glue that allowed shared responsibility to operate effectively, transforming parallel efforts into genuine collaboration.

#### 3.2.2. Navigating Roles and Boundaries

This theme reflects how collaboration was shaped by ongoing negotiation of roles and boundaries. Participants described persistent uncertainty about where professional responsibility ended and family responsibility began. Roles often overlapped or shifted in response to patient condition, clinic workload, and caregiving capacity. This ambiguity occasionally enabled flexibility but more often generated tension and emotional strain.

Nurses operated within institutional expectations as clinical authorities, yet daily practice required reliance on families for ongoing care. Caregivers described being drawn into quasi-clinical roles without feeling adequately prepared or formally acknowledged. Navigating these blurred boundaries was continuous, shaped by communication practices, cultural norms, and the realities of primary care.

1. Unclear Expectations and Role Overlap

Participants frequently described unclear expectations regarding who was responsible for specific aspects of CHF management. Nurses reported that while they provided education and treatment plans, they often assumed families would implement and monitor these plans at home without explicit discussion of limits or contingencies.

*“We explain the plan, but we don’t always say clearly who should do what, especially when the condition changes.”*

(N05)

Family caregivers echoed this ambiguity, describing situations in which they gradually took on increasing responsibility, such as adjusting routines, monitoring symptoms, or deciding when to seek help, without formal guidance or reassurance.

*“At first, I was just helping him take the medicine. Later, I was deciding when to bring him to the center. No one told me where my role stops.”*

(C10)

Role overlap sometimes resulted in duplication of effort or, conversely, in gaps in care when each party assumed the other was responsible. Nurses expressed concern when families independently modified medication schedules or delayed clinic visits, while caregivers described uncertainty about whether they were “overstepping” by questioning instructions or making suggestions.

*“Sometimes the family changes things without telling us, and we only discover it later.”*

(N02)

These situations generated frustration on both sides and highlighted how collaboration without clearly articulated expectations could inadvertently compromise care quality and caregiver confidence.

2. Balancing Professional Authority and Family Knowledge

A second, closely related challenge involved balancing professional authority with recognition of family knowledge. Nurses described holding clinical responsibility and accountability, yet acknowledged that family caregivers possessed intimate, longitudinal knowledge of the older adult’s daily functioning, preferences, and subtle symptom changes.

*“The family sees the patient every day. They notice things we don’t see in the clinic.”*

(N09)

When nurses actively invited caregiver input and validated their observations, collaboration was strengthened and caregivers felt empowered to participate meaningfully.

*“When the nurse asks me what I notice at home, I feel my role is important, not just extra.”*

(C01)

However, participants also described moments where professional authority dominated interactions, particularly in time-pressured consultations. Caregivers reported feeling hesitant to question decisions or share concerns when nurses appeared rushed or when communication felt one-directional.

*“I don’t want to sound like I’m teaching the nurse. So sometimes I keep quiet, even if I’m worried.”*

(C11)

Nurses acknowledged that maintaining authority while remaining open to family input was challenging, especially within rigid clinical workflows that prioritized efficiency over dialogue.

*“We are trained to lead the plan, but collaboration means stepping back sometimes, and that’s not always easy.”*

(N06)

Balancing authority and family knowledge therefore emerged as a delicate, relational process rather than a fixed stance. When successfully negotiated, it fostered mutual respect and shared decision-making; when misaligned, it reinforced hierarchical dynamics that limited caregiver engagement and weakened collaborative care.

#### 3.2.3. Communication as the Engine of Collaboration

This theme captures how communication functioned as the central mechanism enabling or undermining collaboration. Participants framed communication not as a single exchange but as an ongoing process connecting clinical guidance with everyday caregiving realities. When communication was clear, responsive, and continuous, collaboration felt supportive. When fragmented, rushed, or overly technical, it weakened, leaving caregivers uncertain and nurses concerned about adherence.

Communication demands in CHF care were intensified by the complexity of the condition, the advanced age of patients, and the need for sustained self-management beyond clinic walls. Effective collaboration depended not only on what information was shared, but on how, when, and with whom it was communicated.

1. Information Exchange and Education Gaps

Participants highlighted that information exchange and education were foundational to collaborative care, yet often uneven in practice. Nurses described providing education about medications, diet, fluid restriction, symptom monitoring, and warning signs, but acknowledged that time constraints and competing priorities sometimes limited depth and reinforcement.

*“We explain many things in one visit, medicine, salt, fluids, but it’s a lot for families to absorb at once.”*

(N07)

Family caregivers frequently described feeling overwhelmed by the volume and complexity of information, particularly when explanations relied on medical terminology or were delivered rapidly. Some caregivers reported leaving clinic visits with unresolved questions, unsure whether they had fully understood instructions.

*“Sometimes they explain, but very fast. I nod, but later at home I realize I’m not sure.”*

(C05)

Education gaps were especially evident during transitions, such as medication changes or worsening symptoms, when caregivers needed timely, practical guidance. Several caregivers described learning through experience rather than instruction, which increased anxiety and fear of making mistakes.

*“No one told me clearly what to do if his legs swell more. I learned only after it happened.”*

(C03)

Nurses recognized these gaps and expressed frustration when inadequate education compromised home management, yet acknowledged that systemic pressures often constrained their ability to provide individualized teaching. As a result, information exchange was sometimes transactional rather than dialogic, limiting opportunities for clarification and shared understanding.

2. Accessibility and Continuity of Contact

Beyond content, accessibility and continuity of contact emerged as critical enablers of collaboration. Caregivers described feeling more confident and supported when they could reach nurses between visits or see the same nurse consistently over time.

*“When I know who to call, I feel safe. Even if I don’t call, just knowing helps.”*

(C08)

Nurses similarly emphasized that continuity allowed them to build relationships, recognize patterns, and respond more effectively to family concerns.

*“When we see the same family again and again, communication becomes easier. We understand each other.”*

(N10)

However, participants also described challenges related to limited accessibility, high patient volumes, and staff rotation within PHCCs. Caregivers reported uncertainty about whom to contact when concerns arose, sometimes delaying help-seeking or relying on emergency services instead of primary care.

*“If it’s not clinic day, I don’t know who to ask. So sometimes we just wait.”*

(C12)

Nurses acknowledged that workload pressures and organizational structures limited opportunities for follow-up and proactive outreach, even when they recognized its importance.

*“We want to follow up, but there are many patients and very little time.”*

(N01)

Accessibility and continuity therefore functioned as relational infrastructure for collaboration. When present, they transformed communication into an ongoing partnership; when absent, collaboration became episodic and fragile, increasing uncertainty for families and concern for nurses about the sustainability of CHF management.

#### 3.2.4. Cultural and System Constraints Shaping Collaboration

This theme captures how collaboration was shaped by cultural norms and healthcare system constraints beyond individual relationships. Strong moral expectations regarding family caregiving intersected with structural pressures in primary healthcare delivery, simultaneously motivating family involvement and constraining the sustainability of collaborative practices.

Cultural values and system-level realities actively structured how collaboration was understood and enacted. Participants navigated these forces daily, often without institutional support.

1. Family Obligation and Moral Responsibility

Family caregiving for older adults with CHF was consistently framed as a moral and cultural obligation, deeply rooted in religious values, filial duty, and social expectations within Saudi society. Caregivers described their role as non-negotiable, often using language of responsibility, duty, and accountability rather than choice.

*“He is my father. Of course I take care of him. This is not something you discuss.”*

(C06)

This strong sense of obligation motivated sustained involvement and commitment, even in the face of exhaustion or competing responsibilities. Nurses recognized and often relied upon this cultural norm, viewing families as dependable partners who would “do whatever is needed” to support the older adult at home.

*“The family here will not leave the patient alone. They feel it is their duty.”*

(N04)

However, the moral framing of caregiving also limited caregivers’ willingness to express difficulty, fatigue, or uncertainty. Several caregivers described suppressing their own needs to avoid appearing ungrateful, negligent, or disrespectful, which in turn reduced opportunities for nurses to offer additional support or resources.

*“I don’t want to complain. This is my responsibility, even if it is hard.”*

(C09)

Nurses expressed concern that this silent endurance masked caregiver strain and hindered open dialogue. While family obligation facilitated engagement, it also created an unspoken expectation that families would manage regardless of burden, reinforcing imbalances in collaborative care.

2. Time Pressure and System Fragmentation

Alongside cultural expectations, time pressure and system fragmentation emerged as significant structural barriers to effective collaboration. Nurses described working within high-volume PHCCs where limited consultation time constrained opportunities for in-depth discussion, education, and relational engagement with families.

*“We want to sit and explain, but there are many patients waiting. Time controls everything.”*

(N08)

Family caregivers similarly described clinic encounters as rushed, with little space to ask questions or clarify concerns, particularly during busy clinic sessions.

*“Sometimes there are many people waiting, so you don’t feel comfortable asking more.”*

(C01)

System fragmentation further complicated collaboration, particularly across transitions between primary care, hospital services, and specialist clinics. Nurses reported limited access to updated information about hospital admissions, medication changes, or specialist recommendations, often relying on family reports to fill gaps.

*“We depend on the family to tell us what happened in the hospital. Sometimes the information is incomplete.”*

(N11)

Caregivers described feeling caught between services, acting as informal coordinators without clear guidance or authority.

*“They tell us different things in different places. We try to connect them, but it’s confusing.”*

(C02)

These systemic gaps intensified the caregiving burden and placed additional pressure on collaborative relationships. Nurses felt accountable for outcomes without having adequate structural support, while families assumed coordination roles by default. Together, time constraints and fragmented systems constrained collaboration to short, task-focused interactions, limiting opportunities to build the sustained partnerships needed for complex chronic care.

## 4. Discussion

The central finding of this study is that nurse–family collaboration is characterized by shared responsibility alongside persistent role ambiguity and communication challenges. Collaboration emerged as a dynamic, relational process shaped by trust, negotiated roles, communication, and cultural and system-level constraints, fluid and context-dependent rather than linear or standardized. These findings extend existing CHF literature by foregrounding collaboration as lived practice within primary care, rather than as a prescriptive model or outcome variable.

Importantly, this study makes three distinct contributions to the literature. First, it provides empirical evidence from Saudi primary healthcare, a collectivist context where filial obligation is culturally mandated rather than individually chosen, demonstrating how moral responsibility simultaneously enables sustained family engagement and constrains open communication about caregiver burden. This dual mechanism of cultural obligation has not been adequately captured in prior research from Western or individualist healthcare settings. Second, the findings reveal how system-level fragmentation in Saudi primary care forces families into informal care coordination roles that exceed their preparation and authority, a pattern that compounds role ambiguity in ways distinct from healthcare systems with more integrated chronic care pathways. Third, by simultaneously capturing nurse and caregiver perspectives, this study exposes relational asymmetries, particularly the tension between professional authority and experiential family knowledge that shape collaboration differently in a context where hierarchical deference to professionals coexists with strong family autonomy norms.

The theme “We Are Caring Together” underscores shared responsibility in CHF management. Consistent with prior studies, families functioned as extensions of care beyond the clinic, translating clinical advice into daily action [29]. Nurses’ reliance on family caregivers reflects the complex self-management demands of CHF and the limited capacity of older adults to manage independently [30]. Interpreted through relational coordination theory [23], our findings demonstrate that collaboration was most effective when shared goals, mutual respect, and frequent, high-quality communication were present, that is, when responsibility was perceived as collective rather than delegated. This aligns with family-centered care frameworks that emphasize partnership, respect for family roles, and shared decision-making [31,32]. However, whereas relational coordination theory assumes relatively equal status among collaborators, our findings from the Saudi context reveal that cultural hierarchies and moral obligation introduce asymmetries that the theory does not fully account for. Family caregivers’ sense of duty often sustained engagement even when relational coordination mechanisms (e.g., shared knowledge, mutual respect) were weak, suggesting that in collectivist settings, cultural obligation may function as a parallel driver of coordination alongside, and sometimes in tension with, relational quality.

Trust emerged as the relational foundation of collaboration, enabling open communication and reciprocal reliance. Consistent with earlier research, trust developed through continuity, respectful interaction, and recognition of caregivers’ experiential knowledge [33,34]. When trust was established, caregivers felt empowered to report concerns and nurses felt confident integrating family observations into care planning. Conversely, fragile trust limited disclosure and engagement, consistent with evidence that relational quality influences chronic care outcomes [35]. Trust functioned not as an abstract concept but as a product of everyday clinical interactions shaped by time, attitude, and responsiveness. Viewed through the lens of relational coordination theory, trust operates as both an input to and outcome of high-quality communication; in this study, trust was strongest when shared goals were explicit and communication was bidirectional, and weakest when time pressure reduced interactions to one-directional instruction.

The theme Navigating Roles and Boundaries reveals persistent ambiguity regarding responsibility distribution between nurses and families. Unclear expectations resulted in role overlap, caregiver anxiety, and professional concern about safety [36]. In the Saudi context, strong family involvement further blurred boundaries, as moral responsibility overrode formal role delineation. Professional boundary theory provides a useful lens for interpreting these findings: when the boundaries between professional and lay roles are insufficiently articulated, both parties experience role strain and uncertainty. In this study, the absence of explicit role negotiation, compounded by cultural expectations that families will “do whatever is needed”, meant that caregivers frequently crossed into quasi-clinical territory (e.g., medication adjustment, symptom triage) without the knowledge, authority, or institutional support to do so safely. These findings suggest that collaboration requires not only willingness but also deliberate role negotiation, supported by institutional guidance [37].

Balancing professional authority with family knowledge was a critical relational challenge. Nurses navigated accountability for clinical decisions while recognizing that families possessed longitudinal knowledge of patients’ daily functioning. When family input was acknowledged, collaboration strengthened; when overshadowed by hierarchical communication, caregivers withdrew. This tension mirrors debates around power dynamics in healthcare and the shift from paternalistic models toward participatory approaches in chronic care [38]. In the Saudi context, this dynamic was compounded by cultural deference to medical authority, which made it more difficult for caregivers—particularly those of lower educational backgrounds—to assert their experiential knowledge. Professional boundary theory helps explain this pattern: when the boundary between professional and lay knowledge is rigidly maintained, collaboration becomes asymmetric, with caregivers positioned as passive recipients rather than active partners. Our findings reinforce calls for nursing education that legitimizes family expertise while maintaining professional responsibility.

Communication functioned as the “engine” of collaboration, enabling or constraining partnership depending on its quality, continuity, and accessibility. Education gaps left caregivers uncertain and increased reliance on trial-and-error learning, consistent with evidence linking inadequate caregiver education to poor adherence and higher readmission rates in CHF [39]. Participants emphasized not only the content of education but also its timing, repetition, and tailoring to caregivers’ literacy and emotional readiness. Interpreted through health literacy frameworks [26,27], these findings highlight that effective nurse–family communication requires more than information transfer; it demands attention to caregivers’ capacity to access, understand, and apply health information within the practical constraints of home-based care. In the Saudi context, where some caregivers had limited formal education and where medical terminology was often delivered in a language other than the caregiver’s primary dialect, health literacy barriers compounded the communication gaps identified. This suggests that health literacy-informed educational interventions, incorporating teach-back methods, plain-language materials, and culturally appropriate visual aids, may be particularly beneficial in this population.

Accessibility and continuity of contact further shaped collaborative capacity. Consistent relationships with known nurses enhanced trust and reduced caregiver anxiety, while fragmented contact limited reassurance and coordination [40]. However, structural constraints, high patient volumes, staff rotation, and limited follow-up mechanisms restricted sustained engagement. These findings can be interpreted through relational coordination theory, which posits that coordination quality depends on the frequency, timeliness, and accuracy of communication, all of which require relational infrastructure that continuity provides. Addressing these barriers may require system-level innovations such as dedicated chronic care nurses, structured follow-up pathways, or digital communication supports [41]. In the Saudi primary care context, where PHCCs serve large catchment populations with limited specialist staffing, continuity is particularly fragile, suggesting that structural reform, not merely individual effort, is required to sustain collaborative partnerships.

The final theme highlights how collaboration is embedded within broader cultural and system contexts. Strong norms of family obligation motivated sustained caregiving commitment, consistent with studies from collectivist healthcare systems [42]. However, this also discouraged caregivers from expressing burden or seeking support, masking caregiver strain [43]. This finding can be interpreted through the lens of moral economy theory [44], which posits that caregiving within culturally prescribed kinship networks operates according to unspoken rules of reciprocity and obligation that are distinct from formal care arrangements. In the Saudi context, filial duty, reinforced by religious values and community expectations, created a moral framework in which acknowledging difficulty was perceived as a violation of relational obligation. This moral pressure not only concealed caregiver needs from nurses but also constrained nurses’ ability to assess and respond to burden, as caregivers presented a facade of coping. Nurses’ awareness of these dynamics is essential to creating safe spaces for caregivers to articulate needs without fear of moral judgment.

System-level constraints, time pressure and fragmentation between care sectors further limited collaborative depth. Nurses relied on families to bridge information gaps across services, placing additional responsibility on caregivers [45]. In the Saudi primary care system, where electronic health records are not yet fully integrated across primary, secondary, and tertiary levels, families functioned as de facto information conduits—a role that demands health literacy, organizational capacity, and relational confidence that many caregivers lacked. Collaboration consequently became task-focused and reactive rather than anticipatory and relational.

### 4.1. Implications for Practice and Policy

These findings highlight the need for intentional nurse–family collaboration in CHF management in primary healthcare. Collaboration should not rely on informal expectations or assumed family roles. Nurses should be supported to explicitly discuss caregiving responsibilities, clarify boundaries, and negotiate shared roles with family members, particularly during follow-up visits and periods of clinical change. Informed by professional boundary theory, such role clarification may reduce caregiver uncertainty, prevent role overload, and enhance continuity of care.

Ongoing, caregiver-inclusive education is equally important. Caregivers require repeated, tailored explanations regarding symptom monitoring, medication management, and decision-making thresholds. Drawing on health literacy frameworks, educational interactions should be dialogic, incorporating teach-back methods and plain-language materials to ensure comprehension. Nursing practice should emphasize communication skills that support partnership and trust building in chronic disease management.

At the policy level, caregivers should be recognized as essential partners in primary care. Policies promoting continuity of care, stable nurse assignments, protected consultation time, and improved coordination between primary and secondary care are essential to sustaining collaboration. Where family caregiving is culturally embedded, policy initiatives should integrate caregiver assessment and support into routine primary healthcare services.

### 4.2. Limitations

Several limitations should be considered when interpreting the findings of this study. The research was conducted in governmental primary healthcare centers within Riyadh City, which may limit transferability to rural settings, private healthcare sectors, or regions with different healthcare infrastructures. Additionally, although the study included both nurses and family caregivers, the perspectives of older adults living with chronic heart failure were not explored, limiting insight into triadic collaboration involving patients, families, and healthcare professionals.

Participation was voluntary, which may have resulted in self-selection bias, with participants who were more engaged or reflective about collaboration being more likely to take part. As with all qualitative research, the findings are contextually grounded and not intended for statistical generalization. Additionally, social desirability bias may have influenced participants’ responses, particularly given cultural expectations surrounding caregiving and family responsibility. Nevertheless, the depth of participant narratives and the use of rigorous analytic procedures support the relevance of the findings for similar primary care and cultural contexts.

## 5. Conclusions

This study demonstrates that nurse–family collaboration in CHF management is shaped by shared responsibility, trust, and clear communication, yet constrained by role ambiguity, cultural expectations of family obligation, and systemic pressures within primary healthcare. By applying relational coordination theory, professional boundary theory, and health literacy frameworks, this study moves beyond descriptive findings to show that collaboration in the Saudi primary care context is distinctively shaped by the intersection of moral obligation, hierarchical professional norms, and system fragmentation, dynamics that jointly determine whether nurse–family partnerships function as genuinely coordinated care or as parallel, disconnected efforts.

Strengthening this collaboration requires coordinated action across clinical practice, organizational structures, and health policy. Specifically, role clarity, health literacy–informed communication, continuity of care, and structured caregiver support are needed to enhance the quality, sustainability, and person-centeredness of chronic heart failure care for aging populations.

## Figures and Tables

**Table 1:** Demographic and Professional Characteristics of Participants (*n* = 24).

Participant ID	Role	Age (yrs)	Gender	Relationship/Qualification	Years of Experience/Caregiving	Primary Involvement in CHF Care
N01	Nurse	29	Female	BSc Nursing	5 years (PHC)	CHF follow-up, education
N02	Nurse	34	Female	Diploma Nursing	8 years (PHC)	Medication management
N03	Nurse	41	Male	BSc Nursing	15 years (PHC)	Chronic disease clinic
N04	Nurse	38	Female	BSc Nursing	10 years (PHC)	Patient/family counseling
N05	Nurse	26	Female	Diploma Nursing	3 years (PHC)	Vital monitoring
N06	Nurse	45	Male	MSc Nursing	20 years (PHC)	Care coordination
N07	Nurse	31	Female	BSc Nursing	6 years (PHC)	CHF education
N08	Nurse	49	Female	Diploma Nursing	22 years (PHC)	Medication adherence
N09	Nurse	36	Female	BSc Nursing	11 years (PHC)	Family communication
N10	Nurse	33	Female	BSc Nursing	7 years (PHC)	Lifestyle counseling
N11	Nurse	42	Male	MSc Nursing	18 years (PHC)	Referral management
N12	Nurse	28	Female	Diploma Nursing	4 years (PHC)	CHF monitoring
C01	Caregiver	35	Female	Daughter	2 years	Medication & diet
C02	Caregiver	52	Male	Son	6 years	Appointments, monitoring
C03	Caregiver	46	Female	Wife	5 years	Daily care, symptoms
C04	Caregiver	61	Female	Wife	9 years	Full-time caregiving
C05	Caregiver	39	Female	Daughter	3 years	Medication support
C06	Caregiver	44	Male	Son	4 years	Clinic communication
C07	Caregiver	29	Female	Daughter	1 year	Lifestyle support
C08	Caregiver	58	Male	Brother	7 years	Care coordination
C09	Caregiver	47	Female	Daughter	6 years	Symptom monitoring
C10	Caregiver	68	Female	Wife	8 years	Home-based care
C11	Caregiver	41	Male	Son	5 years	Appointment management
C12	Caregiver	36	Female	Daughter	2 years	Medication reminders

**Table 2:** Themes, Sub-Themes, and Descriptions.

Theme	Sub-Theme	Description
1. “We Are Caring Together”	1.1 Shared Responsibility for Daily Management	Nurses and family caregivers described CHF care as a jointly held responsibility, particularly for medication adherence, symptom monitoring, and lifestyle regulation. Collaboration was strongest when roles were complementary and clearly understood.
	1.2 Trust as the Foundation of Partnership	Mutual trust, built through consistency, respect, and responsiveness, enabled effective collaboration and confidence in shared decision-making.
2. Navigating Roles and Boundaries	2.1 Unclear Expectations and Role Overlap	Participants reported ambiguity around who was responsible for specific aspects of care, sometimes leading to tension, duplication of effort, or unmet expectations.
	2.2 Balancing Professional Authority and Family Knowledge	Nurses navigated the tension between clinical authority and recognizing families’ experiential knowledge of the older adult’s condition and daily realities.
3. Communication as the Engine of Collaboration	3.1 Information Exchange and Education Gaps	Effective collaboration depended on clear, tailored communication; gaps in explanation or follow-up undermined families’ ability to manage CHF confidently at home.
	3.2 Accessibility and Continuity of Contact	Ongoing access to nurses, via clinic visits or telephone follow-up, strengthened collaboration, while limited availability weakened coordination and reassurance.
4. Cultural and System Constraints Shaping Collaboration	4.1 Family Obligation and Moral Responsibility	Strong cultural expectations around filial duty motivated family involvement but also intensified caregiver burden and reluctance to voice difficulties.
	4.2 Time Pressure and System Fragmentation	High patient volumes, limited consultation time, and fragmented services constrained nurses’ ability to engage families meaningfully.

## Data Availability

Summarized data supporting the findings of this study are included in the article. Full interview transcripts are not publicly available due to ethical and confidentiality considerations but may be obtained from the corresponding author upon reasonable request and with appropriate ethical approval.
